# 4284 Development of a Survey Instrument to Predict Uptake of and Adherence to Active Surveillance among Men with Low-Risk Prostate Cancer

**DOI:** 10.1017/cts.2020.383

**Published:** 2020-07-29

**Authors:** Aaron T Seaman, Kathryn L. Taylor, Kimberly Davis, Kenneth G. Nepple, Michelle A. Mengeling, Heather Schacht Reisinger, Richard M. Hoffman

**Affiliations:** 1 University of Iowa Institute for Clinical and Translational Science

## Abstract

OBJECTIVES/GOALS: Active surveillance (AS) is a recognized strategy to manage low-risk prostate cancer (PCa) in the absence of cancer progression. Little prospective data exists on the decisional factors associated with selecting and adhering to AS in the absence of cancer progression. We developed a survey instrument to predict AS uptake and adherence. METHODS/STUDY POPULATION: We utilized a three-step process to develop and refine a survey instrument designed to predict AS uptake and adherence among men with low-risk PCa: 1) We identified relevant conceptual domains based on prior research and a literature review. 2) We conducted 21 semi-structured concept elicitation interviews to identify patient-perceived barriers and facilitators to AS uptake and adherence among men with a low-risk PCa who had been on AS for ≥1 year. The identified concepts became the basis of our draft survey instrument. 3) We conducted two rounds of cognitive interviews with men with low-risk PCa (n = 12; n = 6) to refine and initially validate the instrument. RESULTS/ANTICIPATED RESULTS: Relevant concepts identified from the initial interviews included the importance of patient: knowledge of their PCa risk, value in delaying treatment, trust in urologist and the AS surveillance protocol, and perceived social support. Initially, the survey was drafted as a single instrument to be administered after a patient had selected AS comprising sections on patient health, AS selection, and AS adherence. Based on the first round of cognitive interviews, we revised the single instrument into two surveys to track shifts in patient preference and experience. The first, administered at diagnosis, focuses on selection, and the second, a 6-month follow up, focuses on adherence. Following revisions, participants indicated the revised 2-part instrument was clear and not burdensome to complete. DISCUSSION/SIGNIFICANCE OF IMPACT: The instrument’s content validity was evaluated through cognitive interviews, which supported that the survey items’ intended and understood meanings were isomorphic. In the next phase, we plan to conduct a large-scale prospective cohort study to evaluate the predictive validity, after which it will be available for public research use.

